# Salvage Fractionated Stereotactic Radiotherapy with or without Chemotherapy and Immunotherapy for Recurrent Glioblastoma Multiforme: A Single Institution Experience

**DOI:** 10.3389/fonc.2015.00106

**Published:** 2015-05-15

**Authors:** Shaakir Hasan, Eda Chen, Rachelle Lanciano, Jun Yang, Alex Hanlon, John Lamond, Stephen Arrigo, William Ding, Michael Mikhail, Arezoo Ghaneie, Luther Brady

**Affiliations:** ^1^Philadelphia CyberKnife/Crozer Keystone Healthcare System, Philadelphia, PA, USA; ^2^School of Medicine, Drexel University, Philadelphia, PA, USA; ^3^School of Nursing, University of Pennsylvania, Philadelphia, PA, USA

**Keywords:** recurrent glioblastoma, glioblastoma radiosurgery, glioblastoma stereotactic, salvage stereotactic, glioblastoma multiforme

## Abstract

**Background:**

The current standard of care for salvage treatment of glioblastoma multiforme (GBM) is gross total resection and adjuvant chemoradiation for operable patients. Limited evidence exists to suggest that any particular treatment modality improves survival for recurrent GBM, especially if inoperable. We report our experience with fractionated stereotactic radiotherapy (fSRT) with and without chemo/immunotherapy, identifying prognostic factors associated with prolonged survival.

**Methods:**

From 2007 to 2014, 19 patients between 29 and 78 years old (median 55) with recurrent GBM following resection and chemoradiation for their initial tumor, received 18–35 Gy (median 25) in three to five fractions via CyberKnife fSRT. Clinical target volume (CTV) ranged from 0.9 to 152 cc. Sixteen patients received adjuvant systemic therapy with bevacizumab (BEV), temozolomide (TMZ), anti-epidermal growth factor receptor (125)I-mAb 425, or some combination thereof.

**Results:**

The median overall survival (OS) from date of recurrence was 8 months (2.5–61) and 5.3 months (0.6–58) from the end of fSRT. The OS at 6 and 12 months was 47 and 32%, respectively. Three of 19 patients were alive at the time of this review at 20, 49, and 58 months from completion of fSRT. Hazard ratios for survival indicated that patients with a frontal lobe tumor, adjuvant treatment with either BEV or TMZ, time to first recurrence >16 months, CTV <36 cc, recursive partitioning analysis <5, and Eastern Cooperative Oncology Group performance status <2 were all associated with improved survival (*P* < 0.05). There was no evidence of radionecrosis for any patient.

**Conclusion:**

Radiation Therapy Oncology Group (RTOG) 1205 will establish the role of re-irradiation for recurrent GBM, however our study suggests that CyberKnife with chemotherapy can be safely delivered, and is most effective in patients with smaller frontal lobe tumors, good performance status, or long interval from diagnosis.

## Introduction

The most common and aggressive primary brain malignancy in adults, glioblastoma multiforme (GBM), recurs in over 75% of patients with a median time interval of 8 months (1–3). Stupp et al. established radiotherapy with concurrent temozolomide (TMZ) as the initial treatment paradigm for GBM, however, the most appropriate salvage therapy was not determined (3). Thus far, limited evidence exists to suggest that any particular treatment modality improves survival with recurrence (4). The current standard of care for GBM recurrence is gross total resection followed by adjuvant chemoradiation, but only a select number of patients are healthy enough to endure surgery (5). Retrospective data on surgical resection of recurrent GBM suggest a palliative and local control benefit, without prolonged survival (2, 6, 7). Chemotherapy offers a modest survival benefit for recurrent GBM that improves as newer agents are employed, such as TMZ. While TMZ has proven to be an effective salvage therapy, the alkylating agent’s greatest contribution to survival has been as a radiosensitizer (8–12). Recently, the vascular endothelial growth factor (VEGF) inhibitor, bevacizumab (BEV) has also emerged as an effective systemic treatment for recurrent GBM, replacing TMZ as the standard of care (13–16). However, the combination of BEV with chemotherapy in phase II trials revealed increased toxicity without greater efficacy (17–19). Among the emerging immunologic therapies, (125)I labeled anti-epidermal growth factor receptor (EGFR) 425 murine monoclonal antibody (I-mAb 425) produced promising results in a large prospective single-arm study for newly diagnosed GBMs, but the data for its role in recurrence are inconclusive (20). The role of radiotherapy in recurrent GBM treatment is not clearly defined, although retrospective data suggest that there is an improvement in tumor control without a great impact on survival (21).

Because of its utility in the non-surgical setting and its versatility as either definitive or adjuvant treatment, radiation is the most consistently used modality for GBM. Historically, radiotherapy has been used to treat GBMs in the form of conventional external beam radiation, brachytherapy, stereotactic radiosurgery (SRS, single fraction radiation), and fractionated stereotactic radiation therapy (fSRT, two to five fractions of radiation). Phase III trials have shown no benefit to boosting external beam radiation with brachytherapy (22, 23). An additional phase III study failed to demonstrate that SRS boost followed by external beam radiation could deliver a superior outcome compared to standard fractionated external beam radiation alone (24). SRS/fSRT, however, has proven to be non-inferior to conventional radiation and given its convenience and ability to deliver a highly conformal dose with precision, it is emerging as a favorable treatment for recurrent brain tumors (4). Among the technology equipped to deliver SRS, CyberKnife (Accuray Inc., Sunnyvale, CA, USA) is a system in which a linear accelerator mounted on a robotic arm moves in any direction and angle to align with its target and deliver hundreds of radiation beamlets at higher doses and tighter margins than conventional radiation (25). The precision of such technology is well suited for neuro-oncologic treatment, even in the case of re-irradiation, as described in the literature (4).

Several retrospective studies reported survival results for recurrent GBM treated with either SRS or fSRT, with median survival time from re-irradiation between 5.7 to 14.3 months (median 10 months) (26–38). In a prospective cohort of 31 patients with recurrent GBM, Greenspoon et al. described a median overall survival (OS) of 9 months in patients receiving 25–35 Gy in five fractions and concurrent TMZ (39). We aim to contribute to the survival outcomes of patients with recurrent GBM treated by CyberKnife fSRT and either surgery, chemotherapy, immunotherapy, or some combination thereof. We also want to examine possible pretreatment or treatment factors significant for survival, elaborating on the three patients still alive at the time of this review.

## Materials and Methods

From June 2007 to January 2014, 19 patients with biopsy-proven recurrent GBM were treated at the Philadelphia CyberKnife Center and retrospectively reviewed with Institutional Review Board approval. Inclusion in the study required radiographic evidence of remission with computed tomography (CT) or magnetic resonance image (MRI) following initial treatment, as well as radiographic evidence of recurrence, with or without secondary biopsy. Initial treatment with surgery, radiation, or systemic therapy in any combination was considered. Treatment of recurrence had to include fSRT with CyberKnife, with or without surgery, chemotherapy, or immunotherapy.

Contrast-enhanced CT images with 1.25 mm thickness were used to generate individualized treatment plans and to derive digitally reconstructed radiographs to facilitate alignment for stereotactic treatment. T1- and T2-weighted MRI with gadolinium were three-dimensionally fused with the planning CT and transferred to Multiplan software to delineate target volumes and critical structures. Gross tumor volume (GTV) was the same as clinical target volume (CTV) which included the entirety of an enhancing lesion representing tumor or the surgical cavity if the patient had a reoperation. The planning target volume (PTV) included the CTV with 0–2 mm margins (median 1.25 mm). The dose was prescribed to the 65–77% isodose line (median 73%) at a dose of 18–35 Gy (median 25 Gy) in three to five fractions. The biological equivalent dose (BED) ranged from 28 to 60 Gy (median 37.5 Gy), using an α/β of 10. During treatment and planning CT, the patient wore a custom-made immobilization mask. Orthogonal X-rays of the skull were aligned with radiographs reconstructed from the planning CTs and measurements necessary to bring the images into alignment were conveyed to the treatment table for proper adjustment. Skull tracking was performed every three to five beams throughout treatment delivery for optimal position. A linear accelerator mounted on a robotic arm delivered between 103 and 307 (median 150) non-isocentric beams to irradiate a single target stereotactically.

Patients were typically seen 1–3 months after salvage treatment. A CT, PET, or MRI was ordered at least every 3 months following salvage treatment. Recurrence was defined as an enlarging enhancing mass by MRI or PET/CT. Univariate Cox regression models were used to estimate hazard ratios of prognostic factors and Kaplan–Meier curves were used to illustrate OS. Cox and log-rank tests for statistical significance were used where appropriate.

## Results

### Patients

Thirteen males and six females, median age 56 (29–79) had histologically proven primary GBMs between the years 1999 and 2012, with radiographic evidence of recurrence. One of the patients had a primary grade 2 astrocytoma, which recurred as a GBM and resected at time of recurrence. All but two primary tumors were resected, and all patients received conventional radiation at 54–60 Gy in 28–32 fractions, as well as TMZ-based chemotherapy for their initial treatment. The median time to recurrence was 16 months (2–122), and median Karnofsky performance status (KPS) at recurrence was 80 (40–100). Nine patients had a recursive partitioning analysis (RPA) <5, another nine had an RPA equal to 5 with one patient a score of 6. Sizes of recurrent lesions ranged from 0.9 to 152 cc (mean 36 ± 39.9 cc). Upon recurrence, one lesion was completely excised and three were subtotally resected, two of which had gliadel wafers implanted. BEV-based salvage therapy was employed with 4 patients prior to CyberKnife treatment, and 12 received systemic therapy with either BEV (6), TMZ (4), or both (2) after re-irradiation. Three patients received I-425 mAb injections for their initial GBM, and three received the therapy after fSRT for recurrence, though it was never used as an initial salvage treatment. Each patient was re-irradiated with CyberKnife fSRT. One patient received a second fSRT treatment of 20 Gy in five fractions at a different site (right parietal then right frontal), and another patient received 25 Gy in five fractions to the same site in the left temporal lobe. A third patient was re-irradiated for multiple recurrences to 20 Gy in five fractions at the initial tumor site in the right frontal lobe, as well as to 25 Gy in five fractions and 18 Gy in one fraction in new right temporal and right cerebellar sites, and finally to 25 Gy at the fronto-parietal region for a marginal recurrence several years later. A complete list of patient characteristics can be seen on Table 1.

**Table 1:** **Patient characteristics**.

**Patient characteristics**
Number of patients	19
Median age	55	(28–78)
Males	13	(68%)
Females	6	(32%)
ECOG 0–1	15	(79%)
ECOG 2+	4	(21%)
RPA <5	9	(47%)
RPA ≥5	10	(53%)
Median time to recurrence in mo (range)	16	(2–122)
Mean survival from EoT in mo (range)	11.8	(0.6–58)
Median follow-up in mo	5.3	(0.6–58)
**Location**
Frontal	9	(47%)
Temporal	6	(32%)
Parietal	2	(10.5%)
Occipital	2	(10.5%)
**Initial treatment**
Total resection	7	(37%)
Subtotal resection	8	(32%)
Resection (unknown)	4	(21%)
Conventional RT	19	(100%)
Median initial dose in Gy (range)	60	(54–60)
Systemic therapy	19	(100%)
**Recurrence treatment**
Surgery	3
Systemic therapy	14
Temozolomide	7 (2 before RT, 5 after)	
Bevacizumab	9 (3 before RT, 6 after RT)	
^125^I-mAb 425	6 (3 before RT, 3 after RT)	
**CyberKnife**
Mean CTV in cc (range)	35 ± 40 (0.9–151.7)	
Mean dose (range)	25 ± 4 (18–35)	
Mean dose per fraction (range)	5.3 ± 1.3 (4–10)	

*ECOG, Eastern Cooperative Oncology Group Performance Status; RPA, recursive partitioning analysis for glioblastoma multiforme; mo, months; RT, radiotherapy; Gy, Gray; CTV, clinical target volume; EoT, end of treatment*.

### Survival

**Figure 1:**
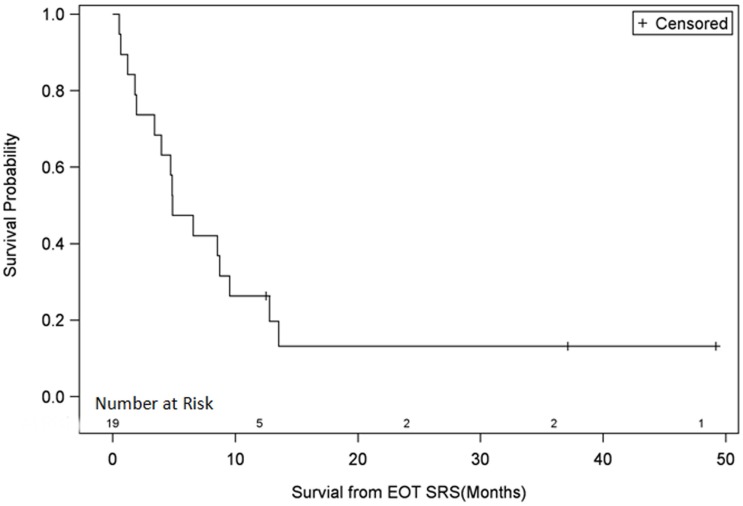
**Survival plot for all patients**. EOT SRS, end of treatment with stereotactic radiosurgery.

The median OS from the date of recurrence for all patients was 8 months (2.5–61) and the median survival from end of fSRT treatment was 5.3 months (0.6–58). The OS of all patients at 3, 6, 9, 12, 24, 36, and 48 months was 74, 47, 32, 26, 13, 13, and 13%, respectively (Figure 1). Three of the 19 patients, who are described in more detail in the discussion, are alive at the time of this review.

**Figure 2:**
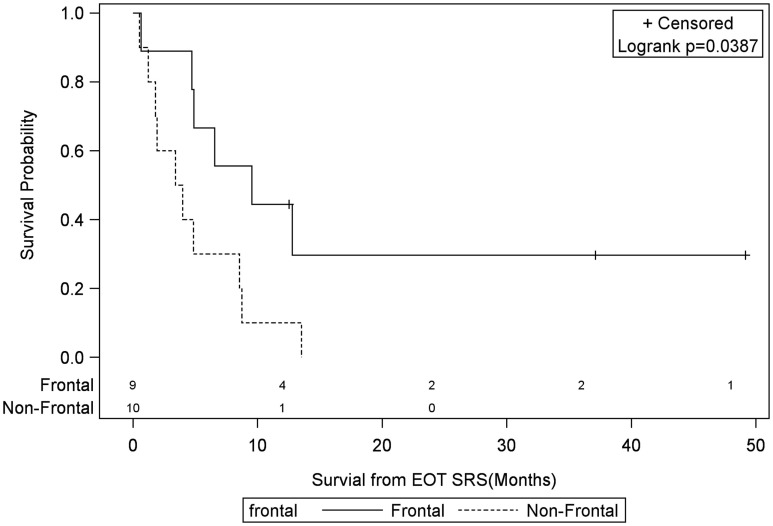
**Months of freedom of death from EOT fSRT by frontal location**. Solid line, frontally located tumor; dotted line, non-frontally located tumor; EOT, end of treatment; fSRT, fractionated stereotactic radiotherapy.

**Figure 3:**
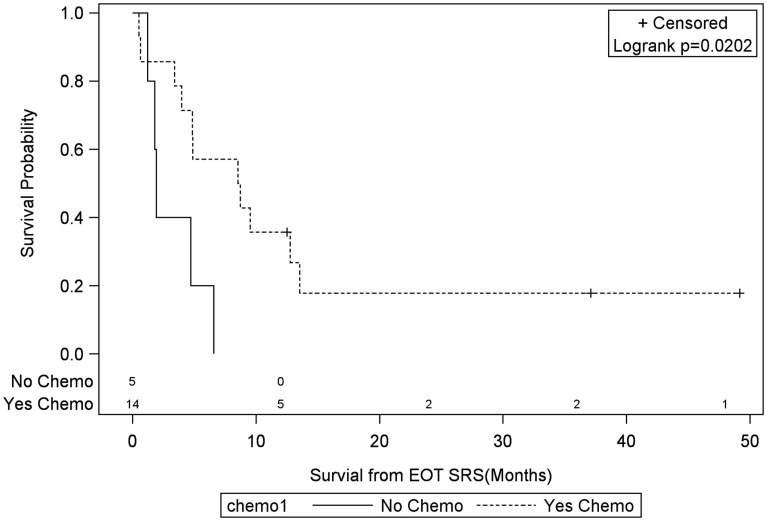
**Months of freedom of death from EOT fSRT by chemo**. Solid line, patients without systonic therapy; dotted line, patients with systemic therapy; EOT, end of treatment; fSRT, fractionated stereotactic radiotherapy.

**Figure 4:**
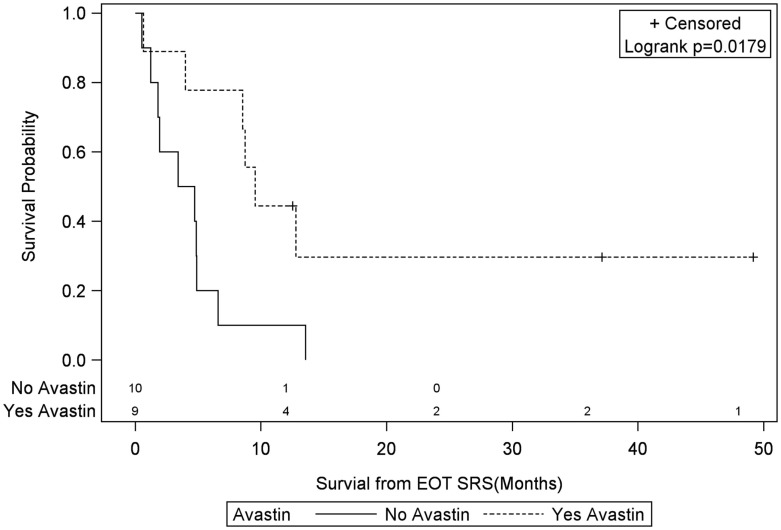
**Months of freedom of death from EOT fSRT by bevacizumab use**. Solid line, patients without bevacizumab; dotted line, patients with bevacizumab; EOT, end of treatment; fSRT, fractionated stereotactic radiotherapy.

**Figure 5:**
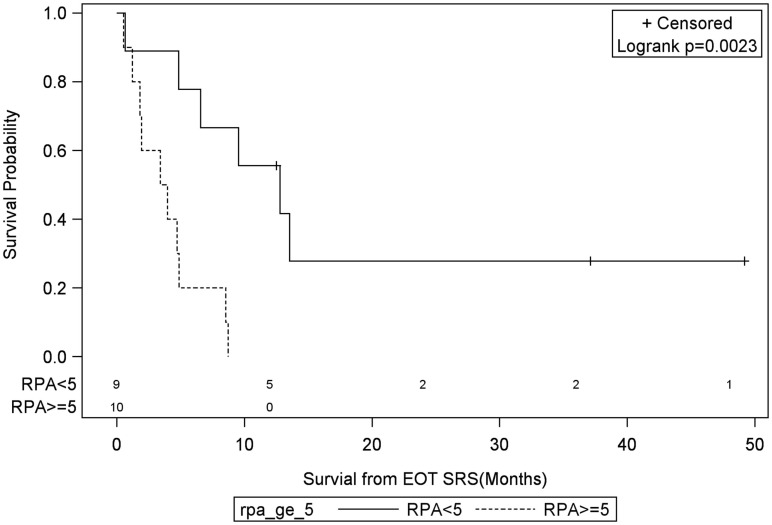
**Months of freedom of death from EOT fSRT by RPA**. Solid line, patients with recursive partitioning analysis less than 5; dotted line, patients with recursive partitioning analysis greater than or equal to 5; EOT, end of treatment; fSRT, fractionated stereotactic radiotherapy.

**Figure 6:**
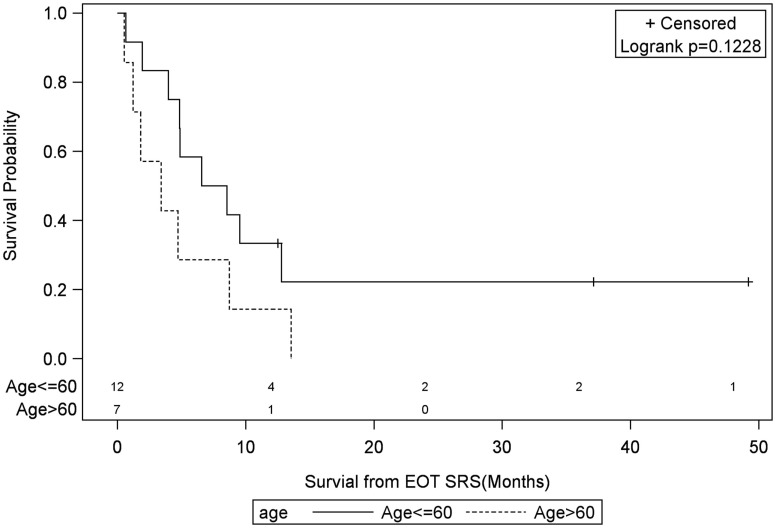
**Months of freedom of death from EOT fSRT by age of recurrence**. Solid line, less than or equal to 60 years old; dotted line, greater than or equal to 60 years old; EOT, end of treatment; fSRT, fractionated stereotactic radiotherapy.

**Figure 7:**
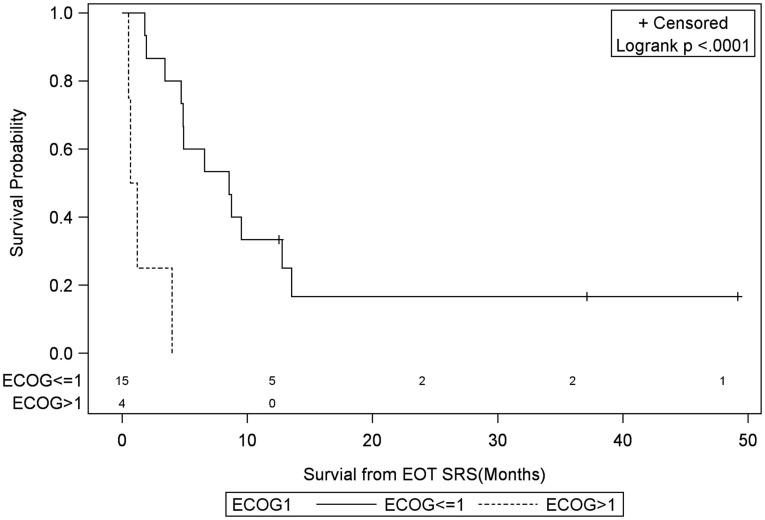
**Months of freedom of death from EOT fSRT by performance status**. Solid line, Eastern Cooperative Oncology Group Performance Status less than or equal to 1; dotted line, Eastern Cooperative Oncology Group Performance Status greater than 1; EOT, end of treatment; fSRT, fractionated stereotactic radiotherapy.

**Figure 8:**
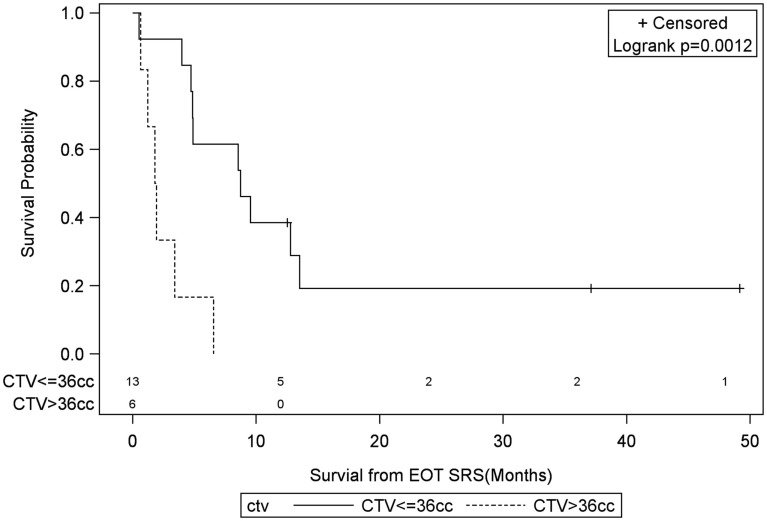
**Months of freedom of death from EOT fSRT by clinical target volume**. Solid line, clinical target volume less than or equal to 36 cc; dotted line, clinical target volume greater than 36 cc; EOT, end of treatment; fSRT, fractionated stereotactic radiotherapy.

**Figure 9:**
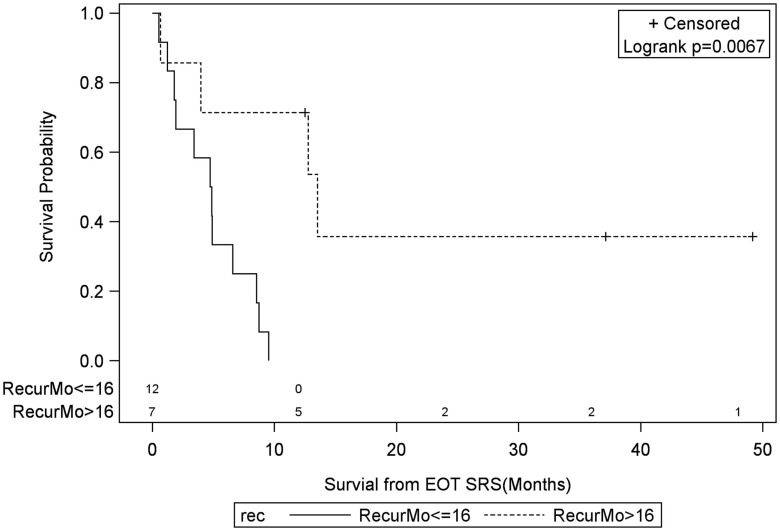
**Months of freedom of death from EOT fSRT by time to recurrence**. Solid line, recurrence less than or equal to 16 months since initial diagnosis; dotted line, recurrence greater than 16 months since initial diagnosis; EOT, end of treatment; fSRT, fractionated stereotactic.

Univariate Cox regression model for survival analysis revealed patients with a frontal lobe tumor (*P* = 0.05), treatment with chemotherapy (*P* = 0.03), treatment with BEV (*P* = 0.03), an RPA <5 (*P* = 0.01), smaller CTVs (*P* = 0.004), a longer interval between initial diagnosis and recurrence (*P* = 0.007), or an Eastern Cooperative Oncology Group (ECOG) performance status <1 (*P* = 0.002) were associated with better survival. Hazard ratios of the aforementioned prognostic factors range from 2.78 (non-frontally located tumor) to 11.8 (ECOG PF >1), all of which are shown in Table 2. Kaplan–Meier regression curves for significant factors are shown in Figures 2–9. KM survival estimates revealed some differences between particular subgroups in mean survival from the end of salvage treatment. Those whose initial tumor recurred after 16 months had a survival of 10.2 months compared to 4.7 months (*P* = 0.007) for tumors recurring sooner. Patients with tumors less than 36 cc survived 8.6 months and those with tumors greater than 36 cc survived 2.6 months (*P* = 0.001). Additionally, mean survival was greater for patients with frontal tumors (8 months) compared to non-frontal tumors (3.3 months, *P* = 0.04) and for those who had salvage chemotherapy (8.6 months) as opposed to those without it (4.9 months, *P* = 0.02).

**Table 2:** **Univariate Cox regression models for overall survival**.

Variable	*P*-value	Hazard ratio
Non-frontal tumor	**0.05**	2.78 (0.99–7.81)
No systemic therapy	**0.03**	3.93 (1.16–13.32)
No bevacizumab	**0.03**	3.31 (1.15–9.58)
RPA ≥5	**0.008**	5.78 (1.57–21.28)
Time to recurrence <16 months^a^	**0.02**	5.69 (1.31–24.81)
ECOG >1	**0.002**	11.8 (2.54–55.16)
CTV >36 cc^b^	**0.004**	6.28 (1.80–21.9)
Age >60	0.42	2.19 (0.81–5.94)

*RPA, recursive partitioning analysis for glioblastoma multiforme; ECOG, Eastern Cooperative Oncology Group Performance Status; CTV, clinical target volume*.

*Bold indicates statistical significance*.

*^a^Median value*.

*^b^Mean value*.

Toxicity was not assessed in this study because of the difficulty in attributing neurocognitive decline to either treatment or cancer progression, especially retrospectively. However, there was no evidence of radionecrosis for any patient following fSRT, nor were any focal deficits noted. Lower grade toxicities such as nausea/vomiting and headache were noted, but not consistently documented.

## Discussion

### Survival

Despite resection being the standard of care for recurrent GBM, median survivals are between 3 and 13 months (40–45), a range comparable to results with radiosurgery. Additionally, several surgical series resulted in negative or insignificant survival differences when compared to patients without reoperation, and up to 40% of patients deteriorated within 3 months following surgery (44, 46). Ideal surgical candidates, those surviving over 10 months, were similar to the long-term survivors in our study: under 60 years old with an ECOG of 0 or 1 and a period of at least 6 months to recurrence (6). Gorlia and Carson et al. examined a pooled group of recurrent GBM patients enrolled in prospective studies receiving conventional chemoradiation with or without surgery, and revealed even more analogous prognostic factors, including prior chemotherapy, frontal tumor location, and tumors less than 50 cc (47, 48).

Retrospective studies similar to this one exhibit 1-year OS rates ranging from 15 (26) to 45% (27) [median 28%, (30)] for recurrent GBM treated with SRS/fSRT, with the wide range most likely attributable to selection bias (Table 3). Two prospective studies, conducted by Larson et al. (28) and Greenspoon et al. (39), had median OSs of 9.5 and 9 months, respectively. Larson’s study included 14 GBM patients who received concurrent chemotherapy and a single fraction of gamma knife SRS prescribed between the 30–40% isodose line, resulting in a median minimal tumor dose of 15 Gy and a median maximum tumor dose of 50 Gy. Greenspoon evaluated 31 patients in which 95% of the PTV received 25–35 Gy in five fractions with concurrent TMZ. The latter study only identified tumor size (<3 cm) as a prognosticator for survival. Greenspoon et al. also reported a grade 3 radiation necrosis rate of 10%, all responsive to steroids and one patient with grade 4 toxicity, responsive to anti-angiogenic therapy.

**Table 3:** **Review of the literature**.

Reference	*N*	Med. dose (range)	No. of Fx	Median BED	Med. size (range)	ReOp rate	Systemic therapy rate	Med. OS from RT (mo)	1-Year OS (%)	2-Year OS (%)
Combs et al. (30)	32	15	1	63.75	10	0	–	7	28	–
Patel et al. (34)	26	18 (12–20)	1	75.6	10 (1–60)	11	–	8.4	–	–
Lederman et al. (37)	88	24	4	43.2	33 (2–50)	12	–	7	17	3.4
Hall et al. (26)	26	20	1	–	28	31	–	7.5	15	0
Shrieve et al. (27)	86	13 (6–20)	1	41.6	10 (2–83)	–	–	10.2	45	19
Mahajan et al. (31)	41	–	1	–	5 (1–16)	–	–	11	29	–
Kong et al. (49)	65	16	1	60.8	–	–	49	13	20.5	–
Larson et al. (28)	14^a^	12–20	1	–	8 (2–30)	–	100	9.5	–	–
Yazici et al. (50)	37	30 (14–32)	1–5	48	24 (2–81)	–	–	10.6	–	–
Martinez et al. (51)	46	18 (14–20)	1	75.6	6	43	–	7.5	40	16
Greenspoon et al. (39)	31^a^	25–35	5	40–56	12	0	100	9	–	–
Current study	19	25 (18–30)	5	40	24	21	74	5.3	26	13

**N*, number of patients; Med, median; No. of Fx, number of fractions; BED, biological equivalent dose; ReOp, reoperation; OS, overall survival; RT, radiotherapy; Mo, months; %, percentage*.

*^a^Prospective study*.

Among the retrospective studies, doses as low as 6 Gy per fraction (37) and as potent as 20 Gy in a single fraction were delivered (26). Normalizing for BED yielded a range of 41.6–75.6 Gy among the various studies, of which higher doses were not associated with longer survival, nor did they report a higher toxicity rate. Upon multivariate and univariate analyses, the most consistent prognostic factor was tumor size (27, 37, 39, 49, 50), with the cutoff volume ranging from 10 cc (27) to 30 cc (37), median 24 cc (50). Youth and performance status were noted as prognostic factors in a few studies (26, 27, 51), while time interval to recurrence, dose, or chemotherapy use were not typically associated with a change in outcome. Our data also suggest that tumor size may be a positive prognosticator, specifically with CTV less than 36 cc, as well as RPA <5. Unlike most of the retrospective series, our results also demonstrated an improvement in survival for tumors located in the frontal region, use of systemic therapy, or longer interval from diagnosis to recurrence of greater than 16 months, but not with age.

### Patients with long-term survival

Three of 19 patients, all males, were alive at last follow-up, who were 58, 55, and 37 years old at diagnosis of recurrence. The first patient was originally diagnosed at age 49 with a grade 2 astrocytoma in the right frontal lobe, which was completely resected, irradiated to 54 Gy with standard fractionation, and recurred as a GBM 10 years later manifesting with left-sided weakness. The 3.2 cm lesion was excised, six Gliadel wafers were implanted in its location. However, treatment-planning MRI 2 months postoperatively revealed enhancement in the surgically resected area, as well as new enhancement in the right temporal lobe and right cerebellum with CTV’s of 5.9, 0.7, and 0.6 cc, respectively. Consequently, in a span of 3 weeks, the original tumor bed was re-irradiated to 20 Gy in five fractions, temporal lesion irradiated to 25 Gy in five fractions, and the cerebellar recurrence received 18 Gy in a single fraction with dose fractionation chosen after review of prior external radiation dose to each site. Three weeks following radiation, the patient had increased mild left-sided weakness that slowly subsided. He had no evidence of disease for almost 4 years, until his performance status declined with frequent falls secondary to left lower extremity weakness. An MRI showed an enhancing lesion posterior to the original tumor, which was once again re-irradiated to 25 Gy in five fractions, resulting in improved motor function of the symptomatic lower extremity. In total, the patient received five separate radiation treatments, four of which were CyberKnife treatments for recurrence. Upon completion of the most recent course of fSRT, the patient completed 12 cycles of BEV with stable disease off any chemotherapy. Although his latest KPS is 50, he is currently alive with no evidence of recurrence at 63 years of age, 4 years and 9 months following initial fSRT.

The second living patient was diagnosed at age 48 with GBM of the left frontal lobe which was completely resected. The patient did not receive postoperative chemoradiation, however he remained free of disease for 5 years. His recurrence was discovered by a follow-up MRI in the left fronto-parietal lobe and was once again resected, this time subtotally. He also received external beam radiation to 60 Gy in 30 fractions to the tumor bed with TMZ. Clinically, the patient had a KPS of 80 with stable right-sided upper and lower extremity weakness and mild motor aphasia, which began when his original tumor was discovered. The patient was maintained on TMZ followed by BEV for 30 months after recurrence until his right-sided weakness became progressively worse, especially in the lower extremity, leading to frequent falls. An MRI showed obvious progression of disease in the left frontal lobe and the patient elected CyberKnife to treat the 9.5 cc lesion with 25 Gy in five fractions. He again received BEV which was stopped over a year ago due to decline in renal function. For over 4 years since fSRT re-irradiation, the patient has shown no evidence of disease progression and although he has a baseline left-sided hemiparesis and mild aphasia, physical and speech therapy has slowly improved those neurological deficits.

The last patient alive at last follow-up was 37 years old when he was originally diagnosed with a frontal butterfly GBM that was subtotally resected followed by 60 Gy of standard external beam radiation and TMZ. He was subsequently given BEV and showed no signs of recurrence until an MRI 2.5 years later showed an increased mass in the genu and rostrum of the corpus callosum. The recurrence was again subtotally resected and adjuvant treatment included 25 Gy in five fractions fSRT re-irradiation with CyberKnife to a suspicious 27.3 cc area near the corpus callosum. Following fSRT, he has been maintained on BEV and irinotecan. At last follow-up, 20 months have passed since completion of fSRT with no evidence of recurrence. Since he was originally diagnosed, the patient has been neurologically asymptomatic with the exception of headaches.

### Limitations

This study is limited by an inherent selection bias given its retrospective nature. The population is relatively heterogeneous with regard to prior treatment and patient characteristics, although not unlike similar studies in the literature. While the data are powered enough for a univariate Cox regression model, a patient population of 19 precludes any type of multivariate analysis. Therefore, the calculated hazard ratios may not reflect the true impact of an associated prognostic factor as covariance likely exists among the variables. However, independent interpretation of a given prognosticator with a significant hazard ratio suggests an effect on survival assuming all other variables are equal.

## Conclusion

Although an improved survival with chemoradiation for inoperable primary GMB patients has been reported, the treatment paradigm for recurrence has not been as clear. However, several studies including this one have demonstrated that SRS/fSRT can be delivered as salvage re-irradiation safely, with survival outcomes comparable to those historically treated with reoperation or chemotherapy alone (6, 11). Furthermore, there may be select patients, particularly those with smaller tumors or good performance status who could potentially benefit from re-irradiation. Our study documents several patients who lived years after re-irradiation via CyberKnife fSRT for recurrent tumors, with favorable prognosticators including frontal lobe location, tumor volume less than 36 cc, use of systemic therapy, or an RPA <5. Confounding variables make it difficult to accurately measure the true impact of such factors on survival, but the data might help provide a starting point for patient selection. Additionally, tumor biology unaccounted for in this experience may also impact survival, such as the presence of radioresistant biomarkers like SYK, STAT3, and SKY pathway genes. In order to investigate which recurrent GBM patients will truly benefit from fSRT/SRS, prospective trials evaluating survival, local control, prognostic factors, and toxicity should be conducted. In the absence of randomized evidence, it remains unknown if radiosurgery improves OS in recurrent GBM, nevertheless it can safely and often times effectively be used as salvage therapy, particularly in conjunction with chemotherapy. Radiation Therapy Oncology Group (RTOG) 1205 should offer valuable insight regarding the efficacy of re-irradiation and BEV vs. BEV alone for recurrent GBM. Although the radiation dose in the phase II trial requires 35 Gy in 10 fractions, which is not considered SRS/fSRT, it may open the door for such prospective trials in the future.

## Conflict of Interest Statement

Dr. Rachelle Lanciano, Dr. Jun Yang, Dr. John Lamond, Dr. Stephen Arrigo, and Dr. Luther Brady each share a small percentage ownership of the Philadelphia CyberKnife Center. The other co-authors declare that the research was conducted in the absence of any commercial or financial relationships that could be construed as a potential conflict of interest.

## References

[1] Wallner KE Galicich JH Krol G Arbit E Malkin MG. Patterns of failure following treatment for glioblastoma multiforme and anaplastic astrocytoma. Int J Radiat Oncol Biol Phys (1989) 16:1405–9. 10.1016/0360-3016(89)90941-3 2542195

[2] Barker FG II Chang SM Gutin PH Malec MK McDermott MW Prados MD Survival and functional status after resection of recurrent glioblastoma multiforme. Neurosurgery (1998) 42:709–20. discussion 720–703, 10.1097/00006123-199804000-00013 9574634

[3] Stupp R Mason WP van den Bent MJ Weller M Fisher B Taphoorn MJ Radiotherapy plus concomitant and adjuvant temozolomide for glioblastoma. N Engl J Med (2005) 352:987–96. 10.1056/NEJMoa043330 15758009

[4] Romanelli P Conti A Pontoriero A Ricciardi GK Tomasello F De Renzis C Role of stereotactic radiosurgery and fractionated stereotactic radiotherapy for the treatment of recurrent glioblastoma multiforme. Neurosurg Focus (2009) 27:E8 10.3171/2009.9.FOCUS09187 19951061

[5] Barbagallo GM Jenkinson MD Brodbelt AR. ‘Recurrent’ glioblastoma multiforme, when should we reoperate? Br J Neurosurg (2008) 22:452–5. 10.1080/02688690802182256 18568742

[6] Brandes AA Bartolotti M Franceschi E. Second surgery for recurrent glioblastoma: advantages and pitfalls. Expert Rev Anticancer Ther (2013) 13:583–7. 10.1586/era.13.32 23617349

[7] Brandes AA Vastola F Monfardini S. Reoperation in recurrent high-grade gliomas: literature review of prognostic factors and outcome. Am J Clin Oncol (1999) 22:387–90. 10.1097/00000421-199908000-00013 10440196

[8] Brandes AA Pasetto LM Monfardini S. New drugs in recurrent high grade gliomas. Anticancer Res (2000) 20:1913–20. 10.1159/000012158 10928126

[9] Brem H Piantadosi S Burger PC Walker M Selker R Vick NA Placebo-controlled trial of safety and efficacy of intraoperative controlled delivery by biodegradable polymers of chemotherapy for recurrent gliomas. The Polymer-brain Tumor Treatment Group. Lancet (1995) 345:1008–12. 10.1016/S0140-6736(95)90755-6 7723496

[10] Chamberlain MC Tsao-Wei DD. Salvage chemotherapy with cyclophosphamide for recurrent, temozolomide-refractory glioblastoma multiforme. Cancer (2004) 100:1213–20. 10.1002/cncr.20072 15022289

[11] Chua SL Rosenthal MA Wong SS Ashley DM Woods AM Dowling A Phase 2 study of temozolomide and caelyx in patients with recurrent glioblastoma multiforme. Neuro Oncol (2004) 6:38–43. 10.1215/S1152851703000188 14769139 PMC1871967

[12] Rajan B Ross G Lim CC Ashley S Goode D Traish D Survival in patients with recurrent glioma as a measure of treatment efficacy: prognostic factors following nitrosourea chemotherapy. Eur J Cancer (1994) 30A:1809–15. 10.1016/0959-8049(94)00248-4 7880611

[13] Chamberlain MC Bevacizumab plus irinotecan in recurrent glioblastoma. J Clin Oncol (2008) 26:1012–3 10.1200/JCO.2007.15.1605 18281677

[14] Kreisl TN Kim L Moore K Duic P Royce C Stroud I Phase II trial of single-agent bevacizumab followed by bevacizumab plus irinotecan at tumor progression in recurrent glioblastoma. J Clin Oncol (2009) 27:740–5. 10.1200/JCO.2008.16.3055 19114704 PMC2645088

[15] Raizer JJ Abrey LE Lassman AB Chang SM Lamborn KR Kuhn JG A phase II trial of erlotinib in patients with recurrent malignant gliomas and nonprogressive glioblastoma multiforme postradiation therapy. Neuro Oncol (2010) 12:95–103 10.1093/neuonc/nop015 20150372 PMC2940554

[16] Vredenburgh JJ Desjardins A Herndon JE II Marcello J Reardon DA Quinn JA Bevacizumab plus irinotecan in recurrent glioblastoma multiforme. J Clin Oncol (2007) 25:4722–9. 10.1200/jco.2007.12.2440 17947719

[17] Verhoeff JJ Lavini C van Linde ME Stalpers LJ Majoie CB Reijneveld JC Bevacizumab and dose-intense temozolomide in recurrent high-grade glioma. Ann Oncol (2010) 21:1723–7. 10.1093/annonc/mdp591 20064829

[18] Reardon DA Desjardins A Vredenburgh JJ Gururangan S Friedman AH Herndon JE II Phase 2 trial of erlotinib plus sirolimus in adults with recurrent glioblastoma. J Neurooncol (2010) 96:219–30. 10.1007/s11060-009-9950-0 19562254 PMC2844073

[19] Sathornsumetee S Desjardins A Vredenburgh JJ McLendon RE Marcello J Herndon JE Phase II trial of bevacizumab and erlotinib in patients with recurrent malignant glioma. Neuro Oncol (2010) 12:1300–10. 10.1093/neuonc/noq099 20716591 PMC3018944

[20] Li L Quang TS Gracely EJ Kim JH Emrich JG Yaeger TE A Phase II study of anti-epidermal growth factor receptor radioimmunotherapy in the treatment of glioblastoma multiforme. J Neurosurg (2010) 113:192–8. 10.3171/2010.2.JNS091211 20345222

[21] Amelio D Amichetti M. Radiation therapy for the treatment of recurrent glioblastoma: an overview. Cancers (Basel) (2012) 4:257–80. 10.3390/cancers4010257 24213239 PMC3712688

[22] Selker RG Shapiro WR Burger P Blackwood MS Arena VC Gilder JC The Brain Tumor Cooperative Group NIH Trial 87-01: a randomized comparison of surgery, external radiotherapy, and carmustine versus surgery, interstitial radiotherapy boost, external radiation therapy, and carmustine. Neurosurgery (2002) 51:343–55. discussion 355–347, 10.1097/00006123-200208000-00009 12182772

[23] Laperriere NJ Leung PM McKenzie S Milosevic M Wong S Glen J Randomized study of brachytherapy in the initial management of patients with malignant astrocytoma. Int J Radiat Oncol Biol Phys (1998) 41:1005–11. 10.1016/S0360-3016(98)00159-X 9719109

[24] Souhami L Seiferheld W Brachman D Podgorsak EB Werner-Wasik M Lustig R Randomized comparison of stereotactic radiosurgery followed by conventional radiotherapy with carmustine to conventional radiotherapy with carmustine for patients with glioblastoma multiforme: report of Radiation Therapy Oncology Group 93-05 protocol. Int J Radiat Oncol Biol Phys (2004) 60:853–60 10.1016/j.ijrobp.2004.04.011 15465203

[25] Lanciano R Lamond J Yang J Feng J Arrigo S Good M Stereotactic body radiation therapy for patients with heavily pretreated liver metastases and liver tumors. Front Oncol (2012) 2:23. 10.3389/fonc.2012.00023 22645716 PMC3355825

[26] Hall WA Djalilian HR Sperduto PW Cho KH Gerbi BJ Gibbons JP Stereotactic radiosurgery for recurrent malignant gliomas. J Clin Oncol (1995) 13:1642–8. 7602353 10.1200/JCO.1995.13.7.1642

[27] Shrieve DC Alexander E III Wen PY Fine HA Kooy HM Black PM Comparison of stereotactic radiosurgery and brachytherapy in the treatment of recurrent glioblastoma multiforme. Neurosurgery (1995) 36:275–82. discussion 282-274, 10.1097/00006123-199502000-00006 7731507

[28] Larson DA Prados M Lamborn KR Smith V Sneed PK Chang S Phase II study of high central dose gamma knife radiosurgery and marimastat in patients with recurrent malignant glioma. Int J Radiat Oncol Biol Phys (2002) 54:1397–404. 10.1016/S0360-3016(02)03743-4 12459362

[29] Kondziolka D Flickinger JC Bissonette DJ Bozik M Lunsford LD. Survival benefit of stereotactic radiosurgery for patients with malignant glial neoplasms. Neurosurgery (1997) 41:776–83. discussion 783-775, 10.1097/00006123-199710000-00004 9316038

[30] Combs SE Widmer V Thilmann C Hof H Debus J Schulz-Ertner D. Stereotactic radiosurgery (SRS): treatment option for recurrent glioblastoma multiforme (GBM). Cancer (2005) 104:2168–73. 10.1002/cncr.21429 16220556

[31] Mahajan A McCutcheon IE Suki D Chang EL Hassenbusch SJ Weinberg JS Case-control study of stereotactic radiosurgery for recurrent glioblastoma multiforme. J Neurosurg (2005) 103:210–7. 10.3171/jns.2005.103.2.0210 16175848

[32] Hsieh PC Chandler JP Bhangoo S Panagiotopoulos K Kalapurakal JA Marymont MH Adjuvant gamma knife stereotactic radiosurgery at the time of tumor progression potentially improves survival for patients with glioblastoma multiforme. Neurosurgery (2005) 57:684–92. discussion 684-692, 10.1227/01.NEU.0000175550.96901.A3 16239880

[33] Cho KH Hall WA Gerbi BJ Higgins PD McGuire WA Clark HB. Single dose versus fractionated stereotactic radiotherapy for recurrent high-grade gliomas. Int J Radiat Oncol Biol Phys (1999) 45:1133–41. 10.1016/S0360-3016(99)00336-3 10613305

[34] Patel M Siddiqui F Jin JY Mikkelsen T Rosenblum M Movsas B Salvage reirradiation for recurrent glioblastoma with radiosurgery: radiographic response and improved survival. J Neurooncol (2009) 92:185–91. 10.1007/s11060-008-9752-9 19066727

[35] Hudes RS Corn BW Werner-Wasik M Andrews D Rosenstock J Thoron L A phase I dose escalation study of hypofractionated stereotactic radiotherapy as salvage therapy for persistent or recurrent malignant glioma. Int J Radiat Oncol Biol Phys (1999) 43:293–8. 10.1016/S0360-3016(98)00416-7 10030252

[36] Vordermark D Kolbl O Ruprecht K Vince GH Bratengeier K Flentje M. Hypofractionated stereotactic re-irradiation: treatment option in recurrent malignant glioma. BMC Cancer (2005) 5:55. 10.1186/1471-2407-5-55 15924621 PMC1156875

[37] Lederman G Wronski M Arbit E Odaimi M Wertheim S Lombardi E Treatment of recurrent glioblastoma multiforme using fractionated stereotactic radiosurgery and concurrent paclitaxel. Am J Clin Oncol (2000) 23:155–9 10.1097/00000421-200004000-00010 10776976

[38] Pouratian N Crowley RW Sherman JH Jagannathan J Sheehan JP. Gamma knife radiosurgery after radiation therapy as an adjunctive treatment for glioblastoma. J Neurooncol (2009) 94:409–18. 10.1007/s11060-009-9873-9 19330482

[39] Greenspoon JN Sharieff W Hirte H Overholt A Devillers R Gunnarsson T Fractionated stereotactic radiosurgery with concurrent temozolomide chemotherapy for locally recurrent glioblastoma multiforme: a prospective cohort study. Onco Targets Ther (2014) 7:485–90. 10.2147/OTT.S60358 24711705 PMC3969344

[40] Ammirati M Galicich JH Arbit E Liao Y. Reoperation in the treatment of recurrent intracranial malignant gliomas. Neurosurgery (1987) 21:607–14. 10.1097/00006123-198711000-00001 2827051

[41] Landy HJ Feun L Schwade JG Snodgrass S Lu Y Gutman F Retreatment of intracranial gliomas. South Med J (1994) 87:211–4 10.1097/00007611-199402000-00013 8115886

[42] Keles GE Anderson B Berger MS. The effect of extent of resection on time to tumor progression and survival in patients with glioblastoma multiforme of the cerebral hemisphere. Surg Neurol (1999) 52:371–9. 10.1016/S0090-3019(99)00103-2 10555843

[43] Pinsker M Lumenta C. Experiences with reoperation on recurrent glioblastoma multiforme. Zentralbl Neurochir (2001) 62:43–7. 10.1055/s-2002-19477 11786935

[44] Mandl ES Dirven CM Buis DR Postma TJ Vandertop WP. Repeated surgery for glioblastoma multiforme: only in combination with other salvage therapy. Surg Neurol (2008) 69:506–9. discussion 509, 10.1016/j.surneu.2007.03.043 18262245

[45] Park JK Hodges T Arko L Shen M Dello Iacono D McNabb A Scale to predict survival after surgery for recurrent glioblastoma multiforme. J Clin Oncol (2010) 28:3838–43. 10.1200/JCO.2010.30.0582 20644085 PMC2940401

[46] Clarke JL Ennis MM Yung WK Chang SM Wen PY Cloughesy TF Is surgery at progression a prognostic marker for improved 6-month progression-free survival or overall survival for patients with recurrent glioblastoma? Neuro Oncol (2011) 13:1118–24. 10.1093/neuonc/nor110 21813511 PMC3177665

[47] Carson KA Grossman SA Fisher JD Shaw EG. Prognostic factors for survival in adult patients with recurrent glioma enrolled onto the new approaches to brain tumor therapy CNS consortium phase I and II clinical trials. J Clin Oncol (2007) 25:2601–6. 10.1200/JCO.2006.08.1661 17577040 PMC4118746

[48] Gorlia T Stupp R Brandes AA Rampling RR Fumoleau P Dittrich C New prognostic factors and calculators for outcome prediction in patients with recurrent glioblastoma: a pooled analysis of EORTC Brain Tumour Group phase I and II clinical trials. Eur J Cancer (2012) 48:1176–84. 10.1016/j.ejca.2012.02.004 22464345

[49] Kong DS Lee JI Park K Kim JH Lim DH Nam DH. Efficacy of stereotactic radiosurgery as a salvage treatment for recurrent malignant gliomas. Cancer (2008) 112:2046–51. 10.1002/cncr.23402 18338759

[50] Yazici G Cengiz M Ozyigit G Eren G Yildiz F Akyol F Hypofractionated stereotactic reirradiation for recurrent glioblastoma. J Neurooncol (2014) 120(1):117–23. 10.1007/s11060-014-1524-0 25012955

[51] Martínez-Carrillo M Tovar-Martín I Zurita-Herrera M Del Moral-Ávila R Guerrero-Tejada R Saura-Rojas E Salvage radiosurgery for selected patients with recurrent malignant gliomas. Biomed Res Int (2014) 2014:657953. 10.1155/2014/657953 24895599 PMC4033521

